# Supplementary material for: The relationship between social rank and spatial learning in pheasants, Phasianus colchicus: cause or consequence?
Source: PeerJ. 2018 Nov 13;6:e5738. doi: 10.7717/peerj.5738 (PMC6238775; doi:10.7717/peerj.5738)
Supplement: Supplemental Information 1 [file peerj-06-5738-s001.docx]

Description of data files to accompany “*The relationship between social rank and spatial learning in pheasants: cause or consequence?*”

Qu. 1. Chick and adult learning performances

- Bird: Individual ID
- Chick X=Final: probability of correct on final trial derived from chick learning curves (20 trials)
- Chick Y=80: predicted trial number when reached 80% probability of choosing correctly, derived from chick learning curves (20 trials)
- Adult X=Final: probability of correct on final trial derived from adult learning curves (20 trials)
- Adult Y=80: predicted trial number when reached 80% probability of choosing correctly, derived from adult learning curves (20 trials)

Qu. 2. Chick learning performances and adult social rank

- Bird: Individual ID
- Chick X=Final: probability of correct on final trial derived from chick learning curves (20 trials)
- Chick Y=80: predicted trial number when reached 80% probability of choosing correctly, derived from chick learning curves (20 trials)
- Mean Elo: mean Elo-rating of Elo-ratings generated from 1000 permuted interaction matrices

Qu. 3. Adult learning performances and adult social rank

- Trial
- Bird: Individual ID
- Correct: 1 yes, 0 no
- Mean Elo: mean Elo-rating of Elo-ratings generated from 1000 permuted interaction matrices
- First choice: correct or incorrect choice on first choice
- Cohort: I or II
- Harem: female group size

Sexual behaviour rates

- Harem: number of females housed with in PD condition
- Male
- Mean Elo-rating
- SG lateral: Social group lateral strut rate
- SG lateral: Perceived dominance lateral strut rate
- Lateral increase: Increase in lateral struts between conditions
- SG crow: Social group crow rate
- PD crow: Perceived dominance crow rate
- Crow increase: increase in crow rate between conditions
